# Teaching Module on Ultrasound-Guided Venous Access Using a Homemade Gel Model for Fourth-Year Medical Students

**DOI:** 10.15766/mep_2374-8265.11222

**Published:** 2022-02-02

**Authors:** Robert James Adrian, April Choi, Sangeeta Lamba, Ilya Ostrovsky, Christine Ramdin, Christin Traba, Sophia Chen, Alexander Sudyn, Stephen Alerhand

**Affiliations:** 1 Fourth-Year Resident, Department of Emergency Medicine, Rutgers New Jersey Medical School; 2 Professor, Department of Emergency Medicine, Rutgers New Jersey Medical School; 3 Assistant Professor, Department of Emergency Medicine, Rutgers New Jersey Medical School; 4 Research Associate, Department of Emergency Medicine, Rutgers New Jersey Medical School; 5 Assistant Professor, Department of Pediatrics, Rutgers New Jersey Medical School; 6 Fourth-Year Medical Student, Rutgers New Jersey Medical School

**Keywords:** Ultrasound Skills, Clinical Skills Assessment/OSCEs, Clinical Teaching/Bedside Teaching, Clinical/Procedural Skills Training, Emergency Medicine, Simulation

## Abstract

**Introduction:**

Evidence supports an ultrasound-guided approach in patients with difficult vascular access. Prior research on teaching ultrasound-guided intravenous access has included only small groups of first- and second-year medical students.

**Methods:**

We enrolled fourth-year medical students in our teaching module. The module featured a 6-minute prelearning narrated lecture and 5-minute orientation, followed by ultrasound-guided IV placement on homemade gel models. Facilitators were emergency medicine (EM) residents with a prespecified level of procedural ultrasound skills according to EM milestones. Students completed pre- and postmodule surveys. Facilitators completed the Directly Observed Procedural Skills Evaluation. Primary outcomes included global rating, proficiency on six procedural skills, and perceived learning.

**Results:**

Our module was completed by 150 fourth-year medical students (94% of the class); 84% cannulated the vein in one attempt. We used a global rating scale to describe the students' cannulation abilities; 59% were trusted to perform this procedure with direct supervision and coaching, 29% with indirect supervision, and 8% without supervision. There was no association between a student's order of attempting IV access within the group and global rating (*p* = .41). Students reported increased understanding of indications, antecubital anatomy, sonographic anatomy, and procedural comfort (12%, 29%, 38%, and 65% improvement pre- vs. postmodule, respectively; *p* < .001).

**Discussion:**

Our module enabled more than one-third of fourth-year medical students to achieve an indirect supervision or better level of proficiency in ultrasound-guided IV access, with significant improvements in perceived knowledge. This module may be useful for other educators facilitating the transition to residency.

## Educational Objectives

By the end of this activity, learners will be able to:

1. List the indications for ultrasound guidance in acquiring peripheral venous access.
2. Describe the venous anatomy in the antecubital fossa and medial upper arm.
3. Identify the venous anatomy using ultrasound.
4. Demonstrate proper technique for acquiring peripheral venous access using ultrasound guidance.
5. Demonstrate proficiency in ultrasound-guided peripheral venous access.

## Introduction

The utility of ultrasound-guided peripheral venous access for adult and pediatric patients with difficult intravenous (IV) access has been clearly demonstrated. Ultrasound-guided IV cannulation provides better odds of success, faster access, fewer attempts and skin punctures, and higher patient satisfaction, as well as decreasing the need for central line placement.^1–7^

Ultrasound-guided IV access can be learned by a variety of learners (nurses, emergency department technicians, and residents) and can lead to improved speed and patient satisfaction.^8,9^ The principles and technique for ultrasound-guided IV cannulation are similar to central vein cannulation under ultrasound guidance, and current guidelines recommend using ultrasound when establishing a central line.^10,11^ Ultrasound is also indicated for patients with obesity, edema, or hypovolemia; who get dialysis; or who have frequently accessed vessels where IV access may be difficult.^5^ Studies have demonstrated that between 8% and 23% of emergency department patients meet criteria for difficult venous access, and in admitted patients with complex health care needs, the prevalence of difficult IV access has been quoted at 59%.^6,12^ Since junior residents are the ones first called for difficult IV access, ultrasound-guided IV placement is a useful skill. Based on these data, an emergency medicine (EM) resident seeing one patient per hour would place 71–351 ultrasound-guided IVs per year.^13^

Placing a standard IV is a basic and core procedural skill per Entrustable Professional Activity (EPA) 12, from the list of procedures medical student should be able to perform prior to graduation.^14^ Establishing IV access could also be considered part of EPA 10, which outlines the emergent care and the initial evaluation and management for which all graduating students should demonstrate basic proficiency.^15^ Though ultrasound-guided IV access is not yet explicitly listed in the EPAs, the skill is routinely required in clinical practice. We believe early exposure to ultrasound-guided IV access will benefit fourth-year medical students pursuing any specialty. We therefore identified ultrasound-guided IV access as an important skill to teach in our transition-to-residency boot-camp course for fourth-year medical students and established this procedure as a mandatory module.

We here describe a curriculum to teach fourth-year medical students how to place ultrasound-guided IVs using a previously described gel model.^16^ A search of *MedEdPORTAL* revealed ultrasound-guided procedure workshops, but none designed for ultrasound-guided peripheral vein cannulation. Other curricula in the literature have taught ultrasound-guided IV cannulation to experienced emergency department staff (e.g., nurses, medical technicians, and residents) or to novices (e.g., first- and second-year medical students) without ultrasound experience.^8,9,17–21^ We focused our effort on an intermediate group of learners, an entire class of fourth-year medical students, as they prepared for internship.

When designing our module, we primarily used the simulation-based procedural training (SBPT) method as it had already shown efficacy in teaching ultrasound-guided procedures.^22^ SBPT allows learners to gain procedural skills in a controlled, low-stakes, standardized, and supervised learning environment that can be designed to accommodate learners' specific needs and assess their skills against standards of achievement.^23^ SBPT is now widely used in medical education in lieu of the traditional “see one, do one, teach one” apprentice model as it reduces harm to patients and has been associated with acquisition of procedural competency by learners.^24^ SBPT follows Kolb's experiential learning theory in which learners observe and analyze a skill being performed (reflective observation and abstract conceptualization) and then formulate a plan for how they themselves will perform the skill before performing it on a task trainer (active experimentation and concrete experience).^24^ The stepwise approach we used aligns well with the four steps of Peyton's teaching approach with demonstration, deconstruction, comprehension, and performance.^25,26^ Additionally, the small-group format incorporates elements of social learning theory, allowing learners to discover new information while working with peers.^24^ To assess procedural performance, we used a validated tool adapted for our needs, the Directly Observed Procedural Skills Evaluation (DOPSE).^27–29^

## Methods

We first obtained approval from the Institutional Review Board of Rutgers New Jersey Medical School (Pro2020000402). We then recruited fourth-year medical students as our learners as part of a 2-week capstone transition-to-residency course. We collaborated with the Office of Education and were able to make this a mandatory learning activity. We assigned students specific time slots on a single day for the practical portion of the module. Three weeks prior (and again the day prior), we sent an informative email to the students. The email contained a 6-minute narrated video lecture and tutorial exposing students to the gel models, IV catheters, handheld ultrasounds, cannulation technique, and expected workflow (Appendix A).

### Setting and Equipment

Students arrived at the clinical skills simulation center, which contained one large conference room and five smaller exam rooms. Each exam room had one patient examination table, one handheld ultrasound device, and two vein gel models (Appendices B and C). We set up rooms with vein gel models, Parker Aquasonic 100 ultrasound transmission gel, standard IV catheters (3 cm, 18G), handheld ultrasound transducers (Butterfly iQ), and iPads/iPhones (see Appendices B and C). Other brands could be substituted without disadvantage. There were multiple handheld ultrasound devices available. We used Butterfly iQ with frequencies between 1 and 10 MHz and scanning depths between 1 and 30 cm. It cost approximately $2,400 per probe, with software and account packages from $430 to $4,400.^30^

### Gel Models

We made 13 (12 with one spare) vein simulators using a homemade ballistic gel model that had been previously described.^16^ We modified these models for our purposes using vinyl tubing and added multiple tube sizes in the gel to represent different vessels to reflect peripheral anatomy. We also used foam earplugs to seal water within the vessels (Appendices D and E). The gels took about 5 hours to create and had to cool for 12 hours.

### Personnel

One EM attending physician with dedicated Clinical Ultrasound Fellowship training and six EM resident facilitators led the module. We established a ratio of one facilitator per five to seven medical students. Formal point-of-care ultrasound training at our residency program involved a 2-week rotation during intern year and third year of residency. Recruited EM residents had achieved graduation targets as determined by the ultrasound division clinical competency committee. The residents had performed a minimum of 150 focused ultrasound examinations and had no prior training in ultrasound teaching. Bearing in mind that procedural competency does not guarantee competency with procedural teaching or assessment, on the day of the module we oriented facilitators to their responsibilities and provided them with the facilitator guide (Appendix F). One EM facilitator produced the ballistic gel model, but the other facilitators were not previously acquainted with it.

### Teaching Activities

We ran six 30-minute sessions for our module, with 25 students at each session (Appendix G). At the beginning of each module, the students were divided into groups of five to seven and led into a small room with an EM resident facilitator. Students were then asked to complete the premodule survey (Appendix H). As the students were expected to have reviewed the study materials emailed to them ahead of time, they were only quickly oriented to the ultrasound transducer, the IV catheters, the iPad display screen, and the steps in performing the procedure. Facilitators held the iPads past the end of the gel and operated them, adjusting the ultrasound gain and depth. The students then began the ultrasound-guided IV cannulation procedure.

### Evaluation

We evaluated students' perceived learning through anonymously completed pre- and postmodule surveys (Appendices H and I). Because the prelearning narrated lecture was part of our module and students took the survey after the lecture but before the DOPSE, the surveys were more precisely pre- and post-DOPSE, but we refer to them here as pre- and postmodule for simplicity. The surveys listed statements regarding the objectives and asked students to agree or disagree using a 5-point Likert scale (1 = *strongly disagree,* 5 = *strongly agree*).

To evaluate each student's proficiency with ultrasound-guided IV cannulation, we created an original DOPSE assessment for each facilitator to complete (Appendix J). Each student was asked to cannulate the vein model with minimal, if any, guidance from the facilitators. Each facilitator completed a DOPSE form for each student within 5 minutes of observing their attempt. Each DOPSE form had six checklist items that were the component parts of the procedure for placing an ultrasound-guided IV, and the facilitator marked whether students were able to achieve each skill independently and without errors. The DOPSE assessment was created for our purposes, based on the relatively well-published Directly Observed Procedural Skills for the education and evaluation of procedural skills, especially in procedure-based specialties.^27–29^ Facilitators also recorded the number of punctures required to cannulate the vein model and the order in which the student had attempted cannulation within their group. Lastly, the facilitators indicated a student's global rating by the degree of supervision each student would need to acquire ultrasound-guided venous access on a patient (“trusted to observe only,” “trusted to perform ultrasound-guided IV placement with direct supervision and coaching,” “trusted to perform with indirect supervision,” “trusted to perform without supervision”).

### Statistical Analysis

Descriptive statistics such as means and proportions were used to summarize the data. The Wilcoxon signed rank test was used to compare the pre- and postmodule surveys.

## Results

A total of 150 fourth-year medical students participated in our module and were evaluated. This constituted 94% of the fourth-year class. The 10 missing students had excused absences.

### DOPSE

Global ratings were as follows: Fifty-nine percent (89 out of 150) were trusted to perform ultrasound-guided IV placement with direct supervision and coaching, 29% (44 out of 150) were trusted with indirect supervision, 8% (12 out of 150) were trusted without supervision, while 1% (1 out of 150) were trusted to observe only. Facilitators did not answer the question in 3% (four out of 150) cases. The differences in these global ratings were statistically significant (*p* < .002) between the groups. We also found that the order in which students completed the procedure within their group did not affect their global rating (*p* = .41; see Table 1 and Appendix J).

**Table 1.**
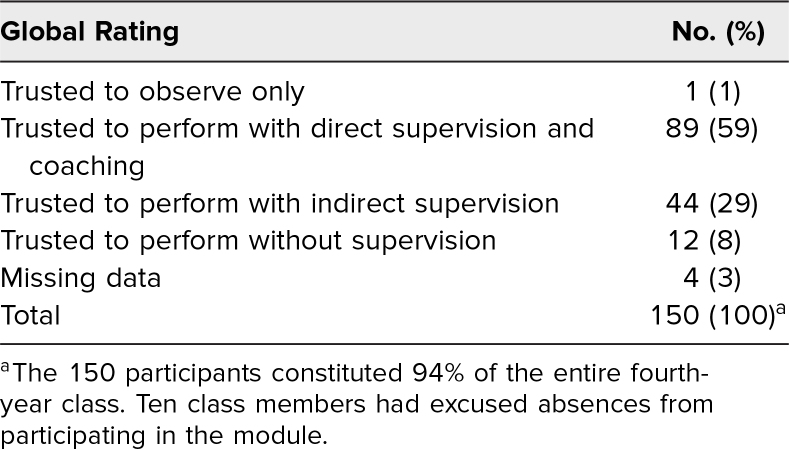
Directly Observed Procedural Skills Evaluation: Global Rating in Ultrasound-Guided IV Access

### Pre- and Postmodule Surveys

There were statistically significant increases in understanding indications, antecubital anatomy, sonographic anatomy, and procedural comfort (*p* < .001; see Table 2 and Appendices H and I).

**Table 2.**
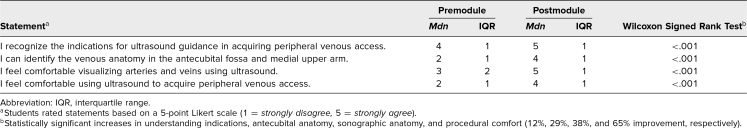
Pre- and Postmodule Surveys: Student Perception and Learning

## Discussion

Peripheral IV access is essential to patient management, and we believe ultrasound-guided venous access has rapidly emerged as a core bedside skill for medical students to acquire prior to entering residency. This skill can also serve as a gateway for other procedures sharing similar principles, such as central line placement. At our institution, medical students are taught how to insert peripheral IVs and introduced to point-of-care ultrasound through preclerkship organ system–based modules. These early exposures allow for familiarity with point-of-care ultrasound as a clinical tool. Our brief module allowed more than one-third of fourth-year medical students to achieve an indirect supervision or better level of proficiency in ultrasound-guided IV access. There were also significant improvements in self-assessed learning and comfort with ultrasound-guided IV access reported by participants.

When assessing the students' skills, we were concerned that students who performed the procedure first in their group might be less successful than those who performed it after watching others. However, we did not find any statistically significant associations between the order in which students performed the procedure and their global rating.

### Lessons Learned

The models worked well in simulating veins, and students were able to successfully visualize the needle tips with point-of-care ultrasound. After a few cannulations, the veins did need to be refilled with water. In addition, the gels were fairly transparent such that the actual vein locations could be partially gleaned with careful intentional study of the model. We addressed this by mounting the display screen using a small iPad stand simulating real-time ultrasound use in the clinical environment. This encouraged students to participate in good faith by compelling their eyes to the screen rather than the gel. High scores on the skills assessment in Appendix J (i.e., “orients the transducer marker properly [left side of screen corresponds with left side of the gel model],” “places ultrasound on the gel model in the transverse plane such that the vein is located on the center of the screen,” “maintains visualization of the needle tip as it advances towards the vessel,” and “visualizes the needle within the vessel in the longitudinal plane”) or on the global rating are not able to be achieved by simply looking at the gel and inserting the catheter without ultrasound guidance. Adding an opaque material, such as a fenestrated paper, artificial skin, or dyes, to obscure the gel may help resolve this issue further.

### Limitations

To better evaluate our entire module, the premodule survey should take place prior to the prelearning narrated lecture. For pragmatic reasons, we had students complete the premodule surveys in person on the day of the practical session. When creating the DOPSE assessment checklist, our primary intention was to devise a scale our instructors could use to evaluate students' placing of an ultrasound-guided IV in our model. We did not evaluate the students' abilities to place ultrasound-guided IVs in real patients. Thus, the level of proficiency as determined by the DOPSE score may not transfer directly to real patients in the clinical setting. There is some literature showing that simulation training in ultrasound-guided vascular access leads to improved clinical performance, but data are limited, and our module would have to undergo its own investigation to evaluate transferability to real patients.^31–33^ Also, the scale in Appendix J has two constructs in place, which undermines the reliability of the instrument.

Furthermore, the true level of proficiency achieved by each student from undergoing this module might not be accurately reflected in their DOPSE score. One reason for this possibility is because we did not formally measure the interrater reliability of our DOPSE evaluations. Evaluation outcomes of the students may also have depended on their previous experiences with ultrasound, instead of being isolated to what they learned from this module. At the time of the module, our institution offered point-of-care ultrasound teaching through optional organ system–based modules. We did not survey students to see if any had volunteered to attend prior modules. Now, point-of-care ultrasound is part of the core curriculum at the first- and second-year medical student levels, so prelecture teaching should be focused on the baseline knowledge of students. We also did not survey students on their previous experience using ultrasound, their prior experience placing IVs, or their prior experience placing ultrasound-guided IVs.

### Future Directions

We plan to repeat this module annually for future classes of students. We have already introduced ultrasound as part of preclerkship organ system–based curricula, and we look forward to seeing how that may affect student proficiency and comfort with this skill. As next steps, we hope to poll students about the potential areas where ultrasound guidance could be utilized in their chosen field of specialization and the relevance of this module to their specialty skill development. We also plan to improve the fidelity of the gel models with simulated skin, colored dyes, and other techniques from the simulation literature. It may be helpful to enroll students in a repeated DOPSE to assess retention of skills.

## Appendices


Ultrasound-Guided Peripheral Venous Access.mp4Practical Session Room Setup.pdfSmall-Room Setup.docxPhoto Deck Directions.pdfItemized Materials for Creating Gel Models.docxFacilitator Guide.docxSchedule.docxPremodule Survey.docxPostmodule Survey.docxDirectly Observed Procedural Skills Evaluation.docx
*All appendices are peer reviewed as integral parts of the Original Publication.*
