# Supplementary material for: Teaching Module on Ultrasound-Guided Venous Access Using a Homemade Gel Model for Fourth-Year Medical Students
Source: MedEdPORTAL. 2022 Feb 2;18:11222. doi: 10.15766/mep_2374-8265.11222 (PMC8807663; doi:10.15766/mep_2374-8265.11222)
Supplement: Supplementary file 1 — Ultrasound-Guided Peripheral Venous Access.mp4Practical Session Room Setup.pdfSmall-Room Setup.docxPhoto Deck Directions.pdfItemized Materials for Creating Gel Models.docxFacilitator Guide.docxSchedule.docxPremodule Survey.docxPostmodule Survey.docxDirectly Observed Procedural Skills Evaluation.docx [file mep_2374-8265.11222-s001.zip › H. Premodule Survey.docx]

**Appendix H: Pre-Module Survey**

| **Please indicate your level of agreement with the following statements**: | **Strongly Disagree**    **1** | **Disagree**      **2** | **Neutral**      **3** | **Agree**      **4** | **Strongly Agree** |
| --- | --- | --- | --- | --- | --- |
| 1. I recognize the indications for ultrasound guidance in acquiring peripheral venous access. |  |  |  |  |  |
| 2. I can identify the venous anatomy in the antecubital fossa and medial upper arm |  |  |  |  |  |
| 3. I feel comfortable visualizing arteries and veins using ultrasound. |  |  |  |  |  |
| 4. I feel comfortable using ultrasound to acquire peripheral venous access. |  |  |  |  |  |

Name: ________________________________
