# Supplementary material for: Teaching Module on Ultrasound-Guided Venous Access Using a Homemade Gel Model for Fourth-Year Medical Students
Source: MedEdPORTAL. 2022 Feb 2;18:11222. doi: 10.15766/mep_2374-8265.11222 (PMC8807663; doi:10.15766/mep_2374-8265.11222)
Supplement: Supplementary file 1 — Ultrasound-Guided Peripheral Venous Access.mp4Practical Session Room Setup.pdfSmall-Room Setup.docxPhoto Deck Directions.pdfItemized Materials for Creating Gel Models.docxFacilitator Guide.docxSchedule.docxPremodule Survey.docxPostmodule Survey.docxDirectly Observed Procedural Skills Evaluation.docx [file mep_2374-8265.11222-s001.zip › C. Small-Room Setup.docx]

**Appendix C: Small Room Setup**

Flow of Events

- In case of potential delays and/or transitions between students, there will be 10-minute breaks in between sessions, along with a 10-minute buffer time at the end (see Schedule below).
- The US Director will serve as “pit-boss.” Faculty will roam around to make sure sessions are running on time, students are going where they are supposed to, technological and equipment issues are addressed, and any other needs are met.
- Another faculty will serve as a second “pit-boss” and step in to run a station as needed (e.g. resident needs a bathroom break).
- One of the two faculty members will run the timer. Faculty will announce a switch every 5 minutes (ex. “Student 1’s time is up”, “Student 2’s time is up”, etc.).
- Faculty will collect the Teaching Objective Structured Clinical Examination sheets from each station at the end of each session.
- Faculty will collect the Post-Module Surveys as the students leave the Clinical Skills Center.

Student Assignments

- Students will be pre-assigned both a letter/time and a room number.
  - A1-5, B1-5, C1-5, D1-5, E1-5, F1-5

Needs Assessment

Each station will include the following:

- 1 table in each room
- 1 chair in each room for facilitator
- 1 time schedule posted on door
- 5-6 Teaching Objective Structured Clinical Examination sheets
- 5-6 Post-Module surveys
- 1 hands-on facilitator
- 1 peripheral intravenous access ballistic gel model
  - The gel model will be switched out and replaced prior to Session D (10 pm)
- 1 blue chuck on which to place the ballistic gel model
- 1 handheld ultrasound
- 1 iPad/display screen
- 1 outlet with corresponding charging cords
- 1 bottle of Aquasonic ultrasound gel
- 1 roll of paper towels for wiping the ultrasound gel
- 35 intravenous catheters (1 per student), with sufficient extras as needed
- 1 large plastic cup holding the 35 intravenous catheters
- 1 large sharps container for discarding used catheters
