# Supplementary material for: Teaching Module on Ultrasound-Guided Venous Access Using a Homemade Gel Model for Fourth-Year Medical Students
Source: MedEdPORTAL. 2022 Feb 2;18:11222. doi: 10.15766/mep_2374-8265.11222 (PMC8807663; doi:10.15766/mep_2374-8265.11222)
Supplement: Supplementary file 1 — Ultrasound-Guided Peripheral Venous Access.mp4Practical Session Room Setup.pdfSmall-Room Setup.docxPhoto Deck Directions.pdfItemized Materials for Creating Gel Models.docxFacilitator Guide.docxSchedule.docxPremodule Survey.docxPostmodule Survey.docxDirectly Observed Procedural Skills Evaluation.docx [file mep_2374-8265.11222-s001.zip › D. Photo Deck Directions.pdf]

1. Gather supplies

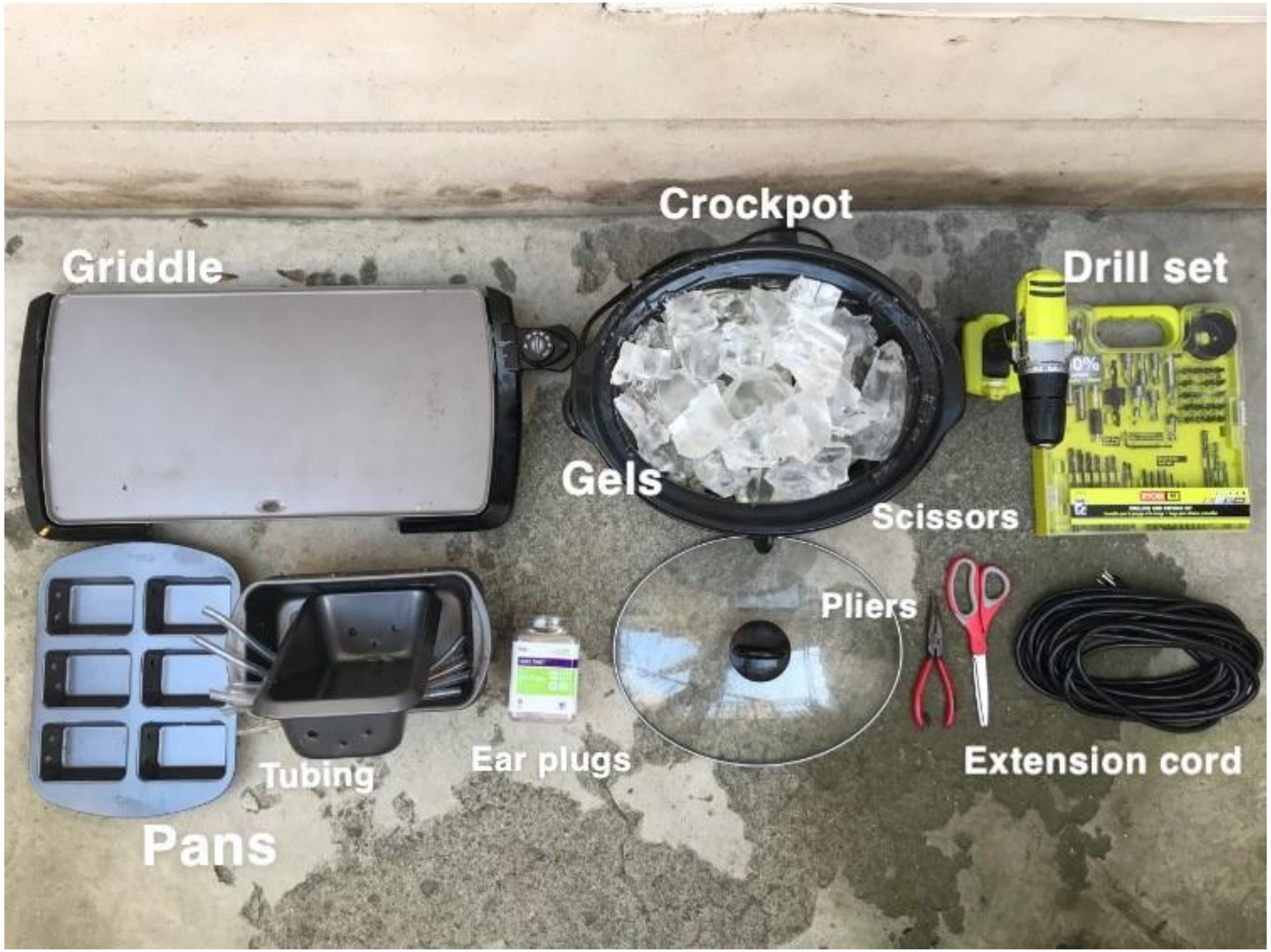

Author-Owned Images

2. Turn slow cooker to low (200-250°F)
3. Place small pieces of ballistic gel into slow cooker

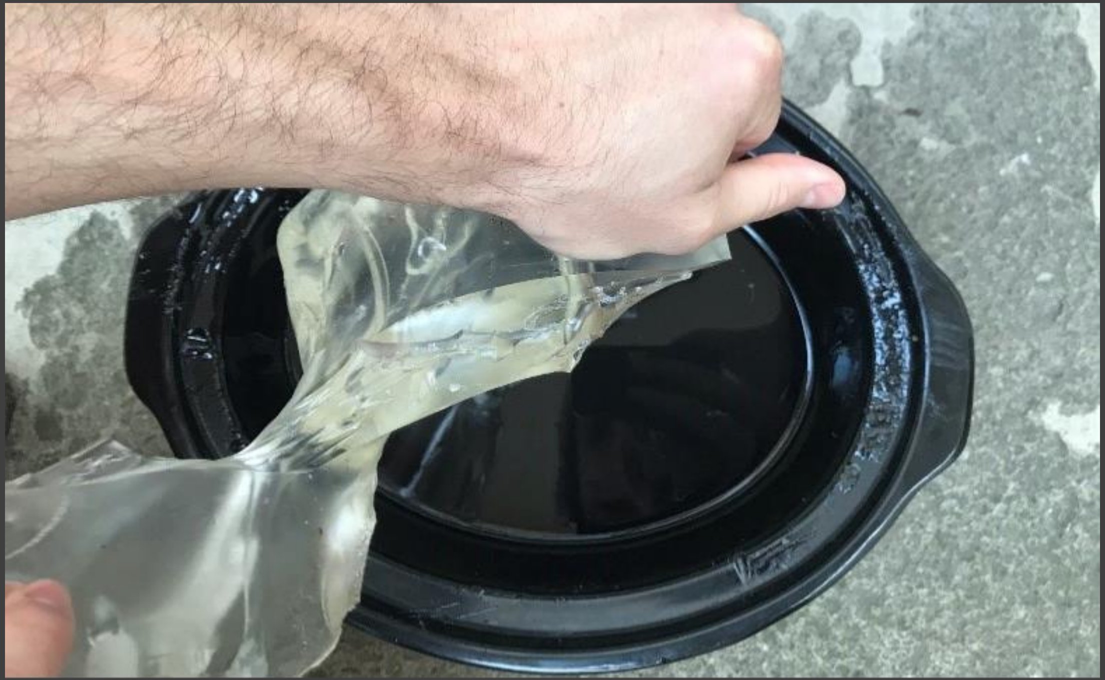

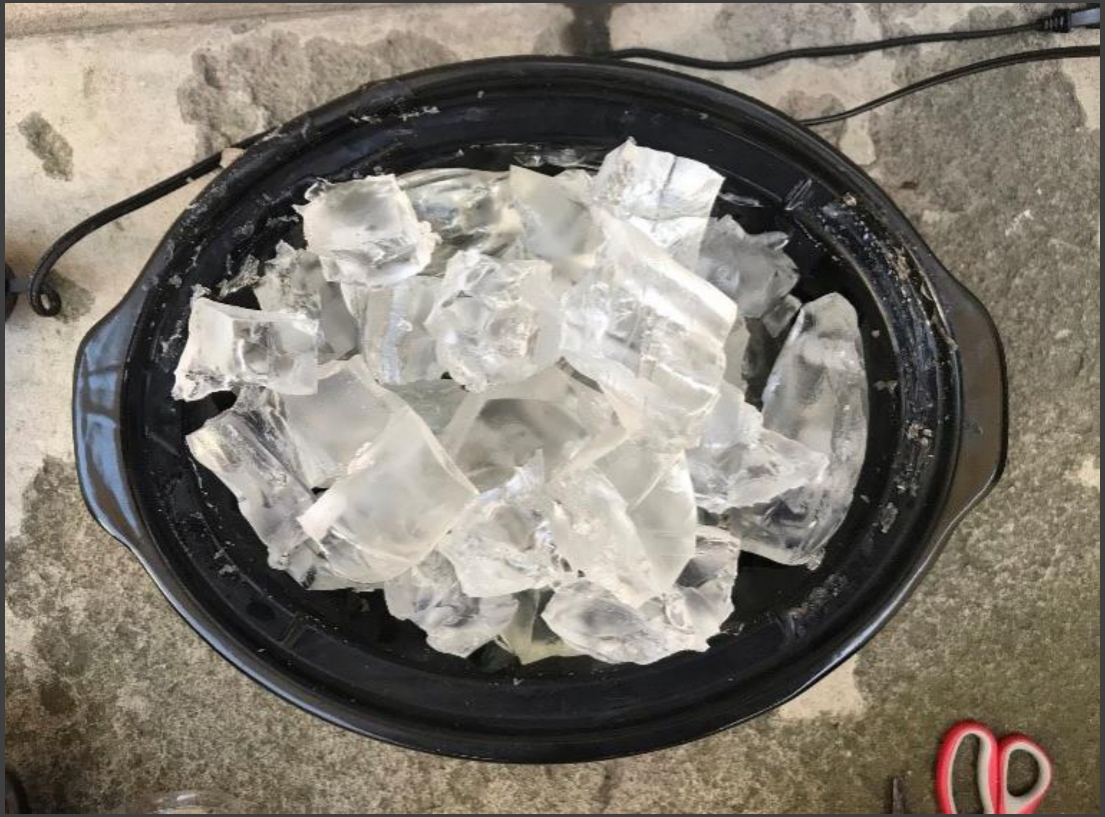

Author-Owned Images

4. Heat until liquified

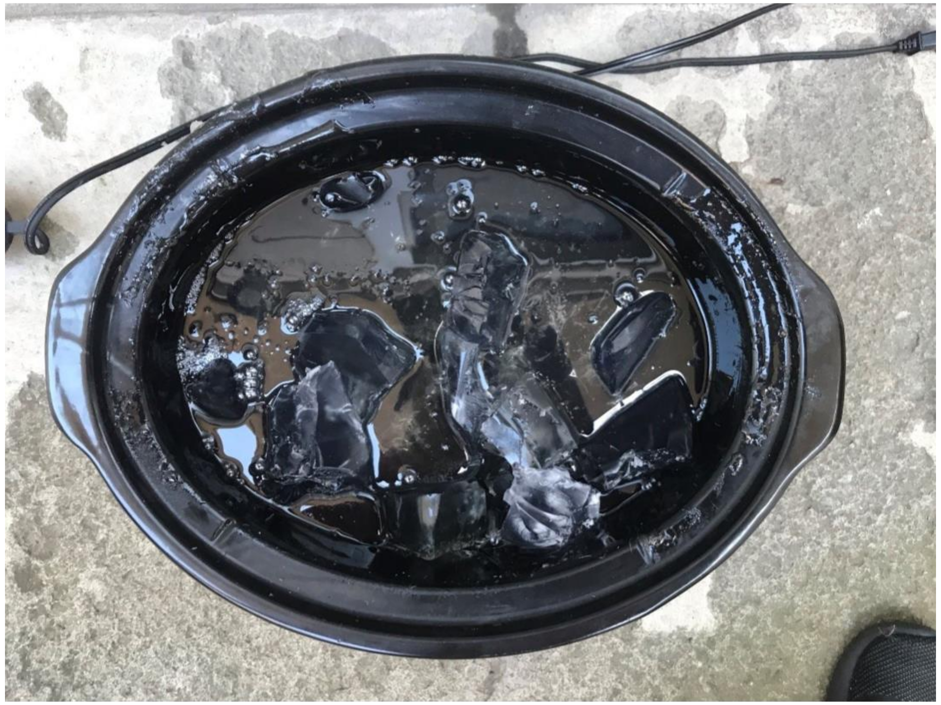

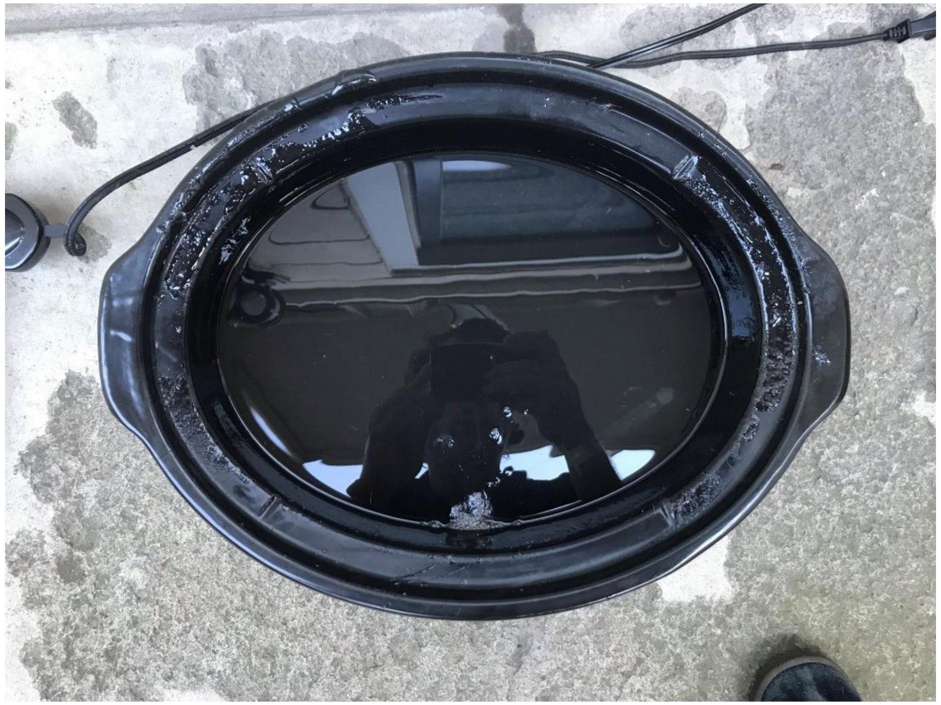

Author-Owned Images

4. While the gel is heating, prepare your pan molds. Choose drill bit the size of the outer diameter of the tubing (¼ in : ¼ in). Wear eye protection. Drill holes centrally.

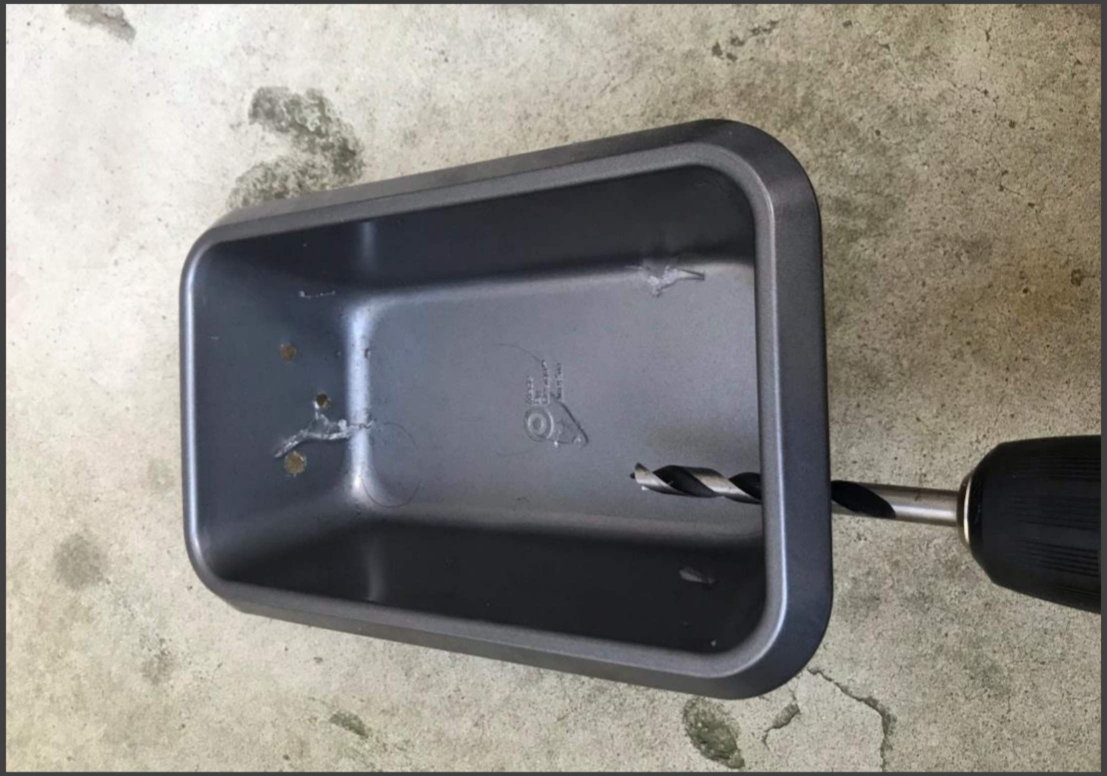

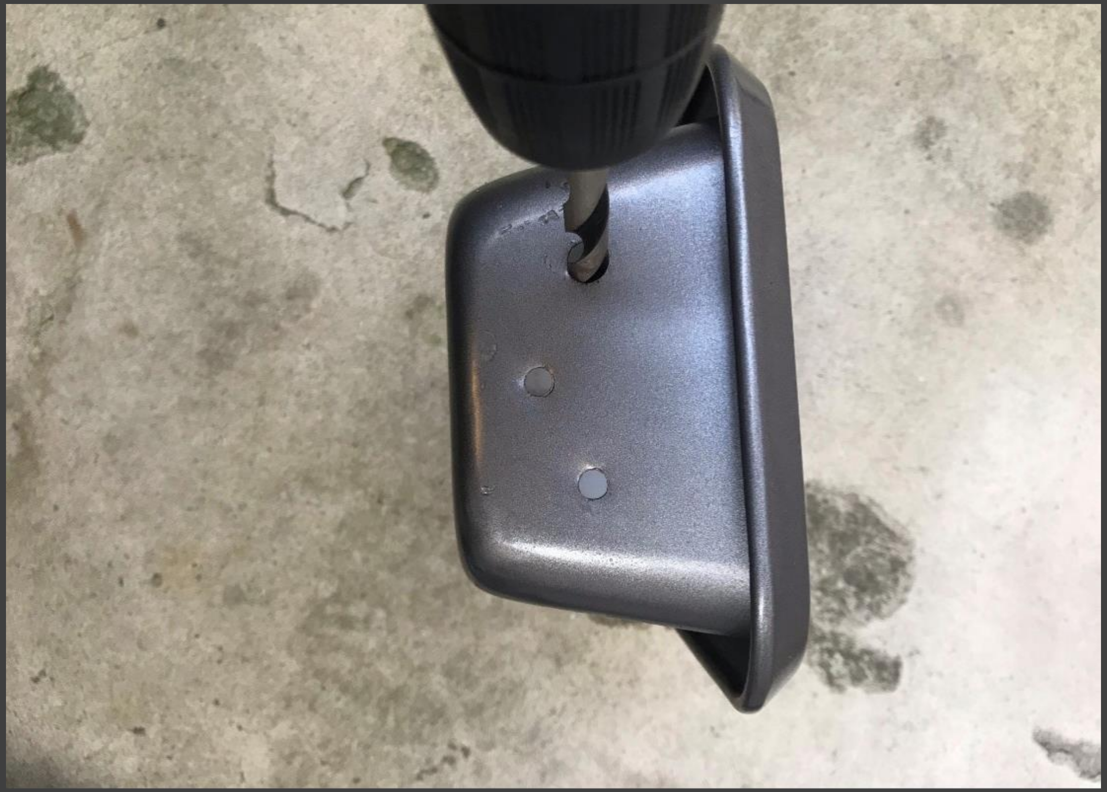

Author-Owned Images

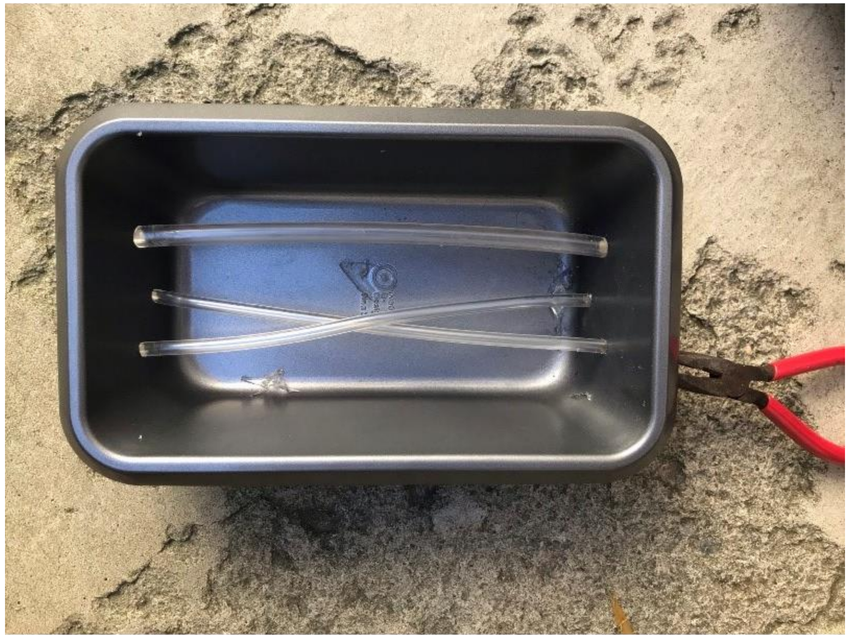

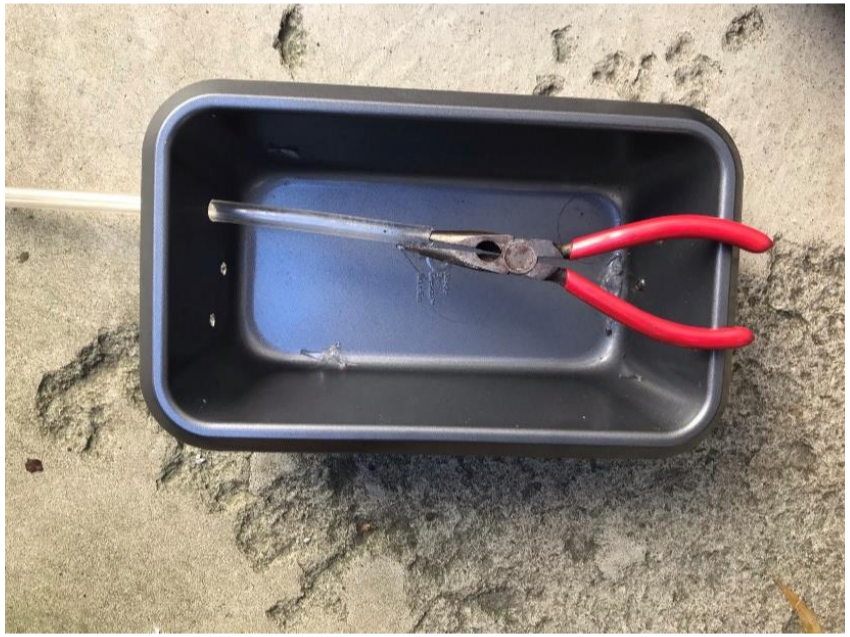

5. Cut tubing on an angle to more easily pull through holes

Author-Owned Images

6. Preheat hot plate to 250-270°F

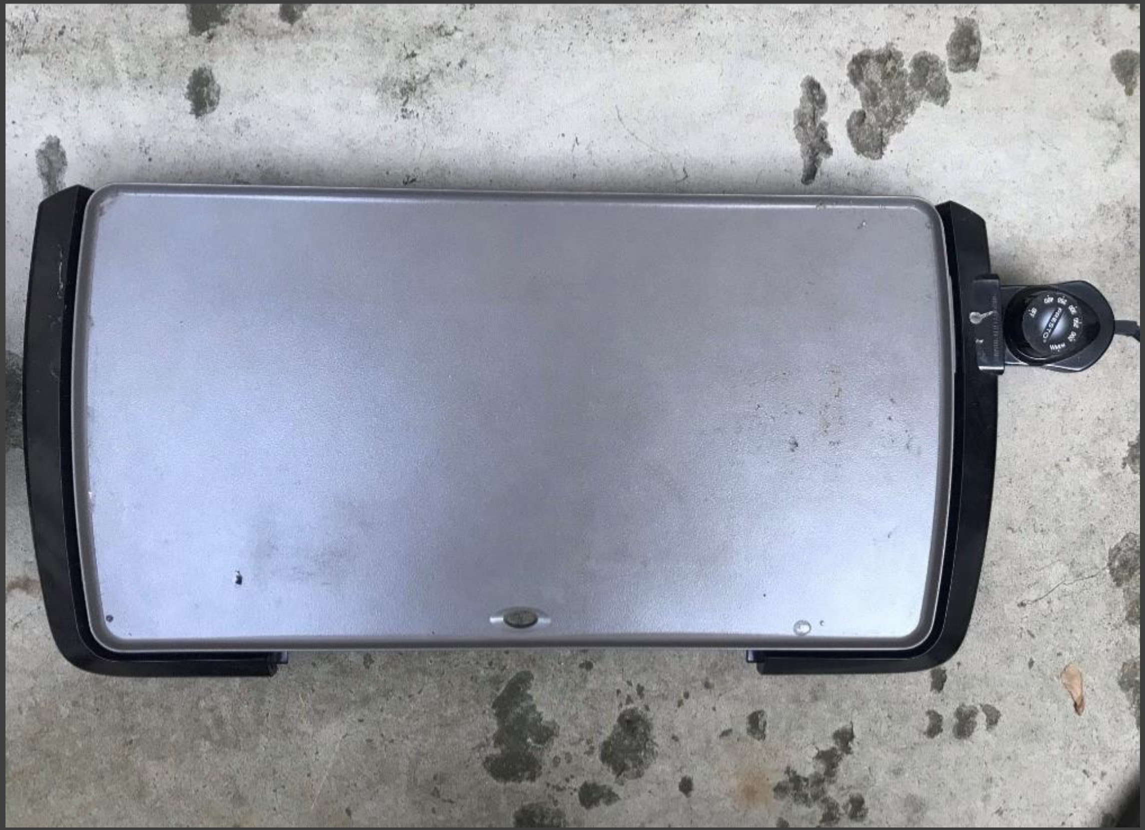

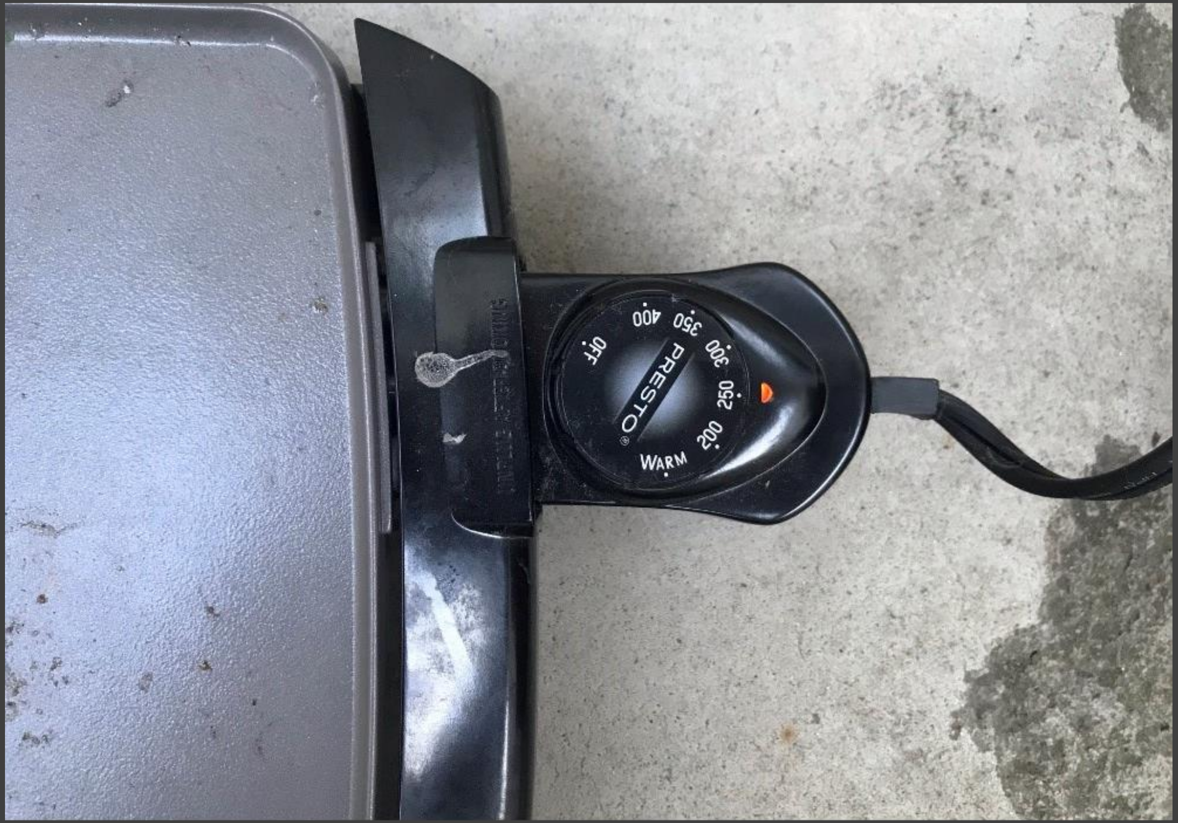

Author-Owned Images

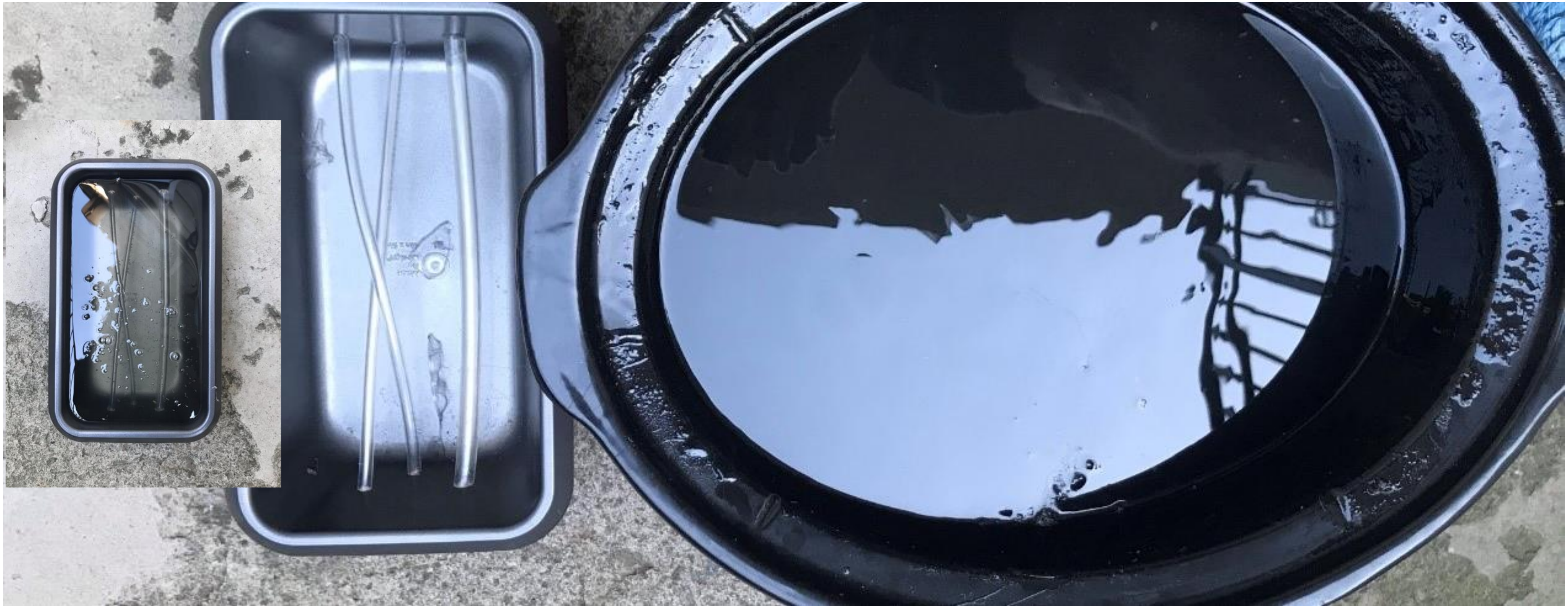

7. Pour hot liquid into pan. Fill about 3 cm above the tubes. Turn off slow cooker.

Author-Owned Images

7. Place pan onto hot plate. Heat so that internal air bubbles escape. Takes about 1 hour.

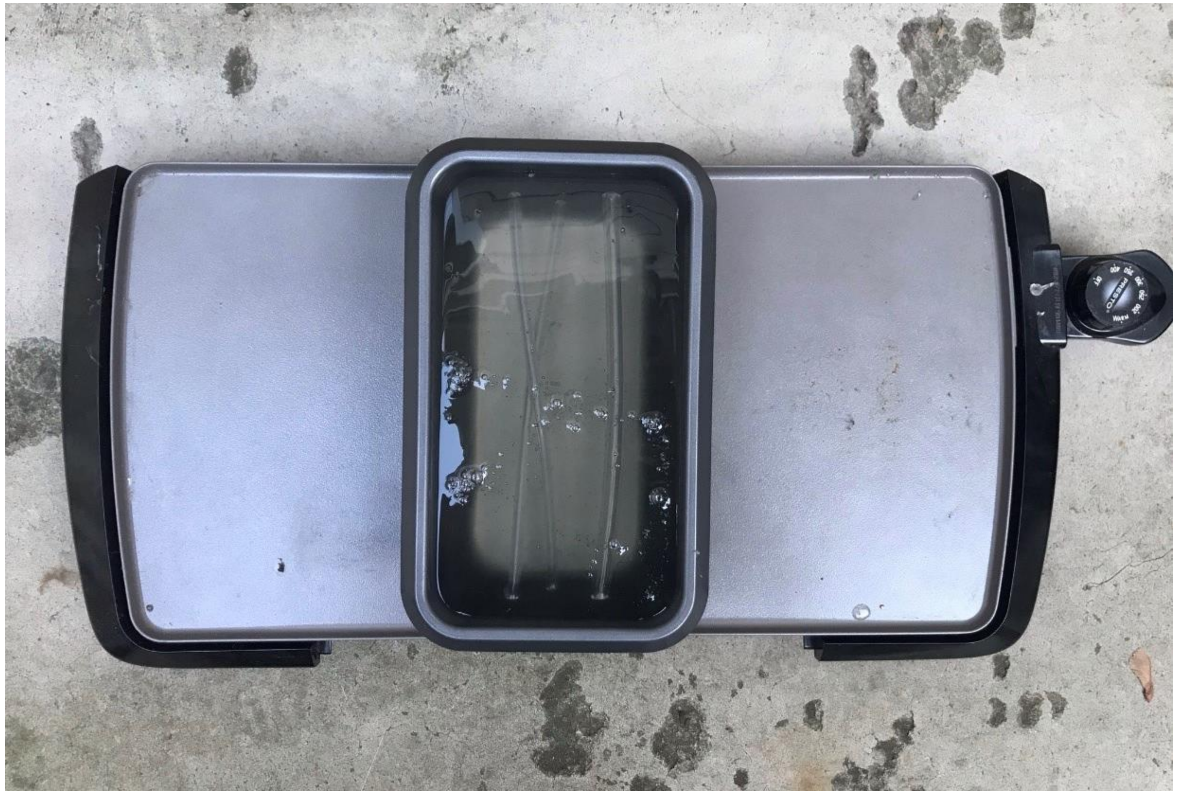

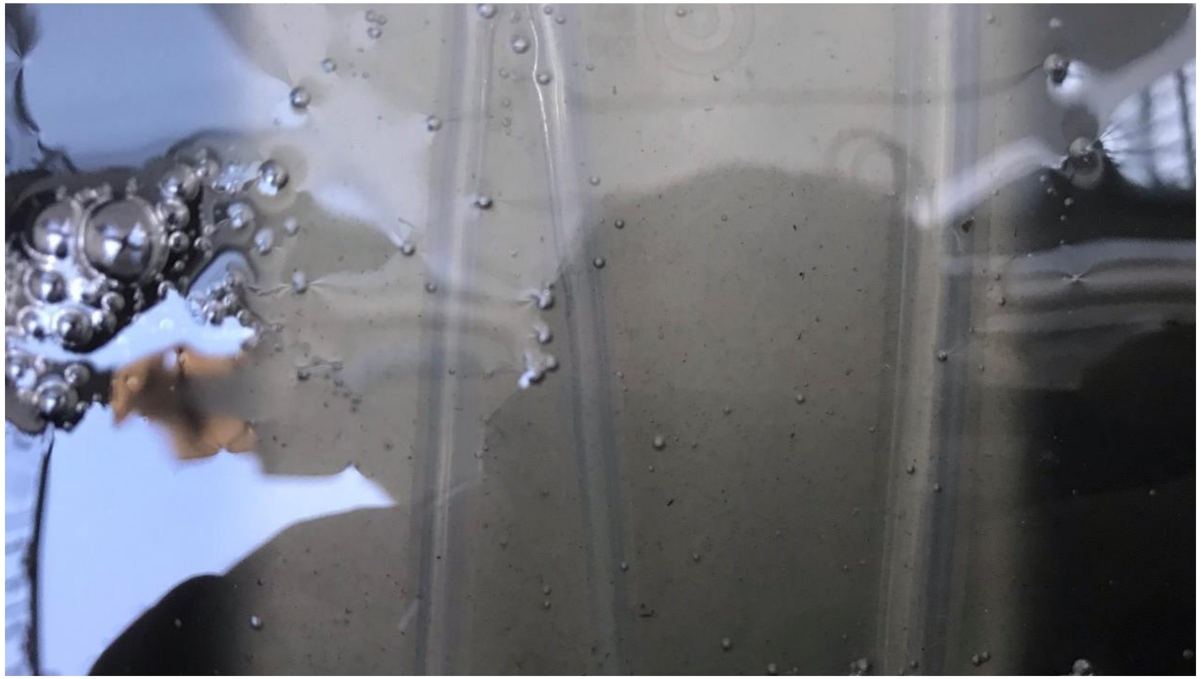

Author-Owned Images

8. Remove pan from hot plate. Turn off hot plate. Ensure internal bubbles have escaped. Cool for 12 hours.

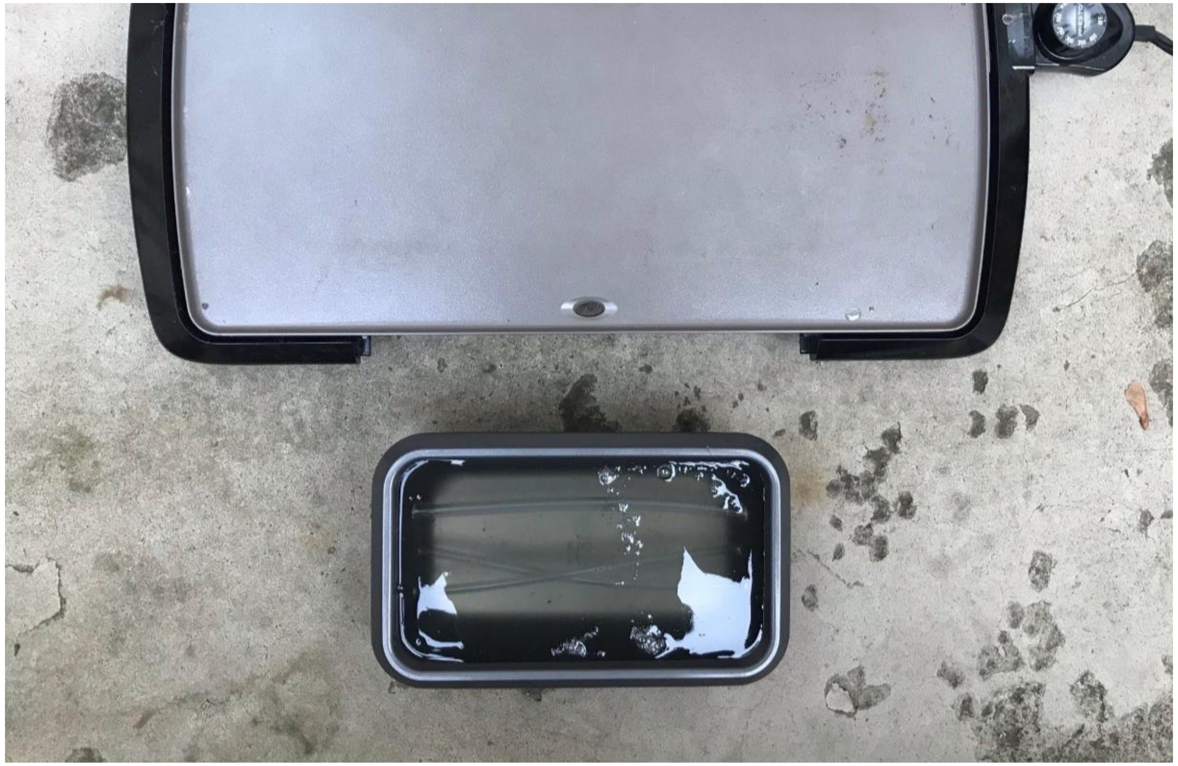

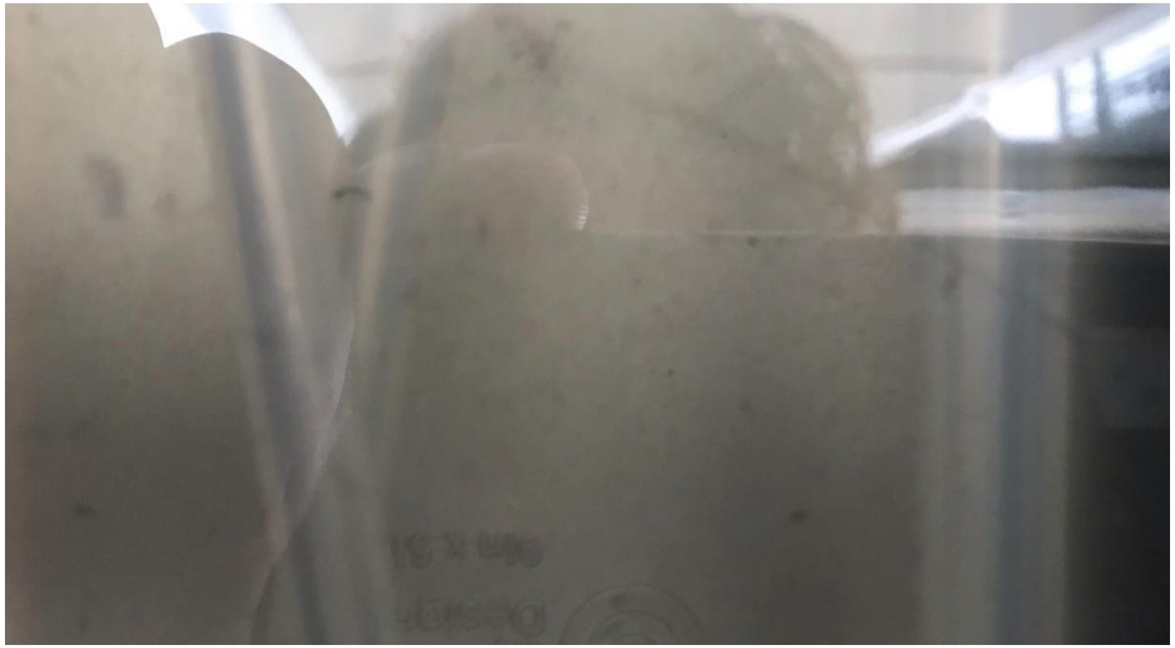

Author-Owned Images

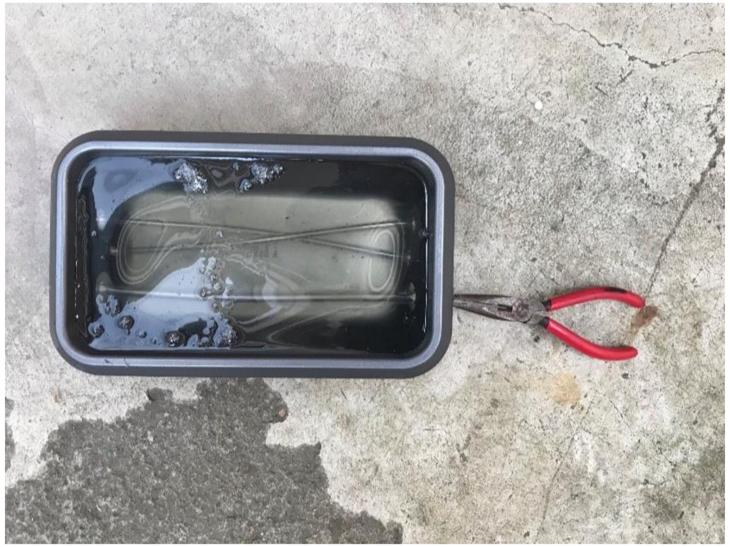

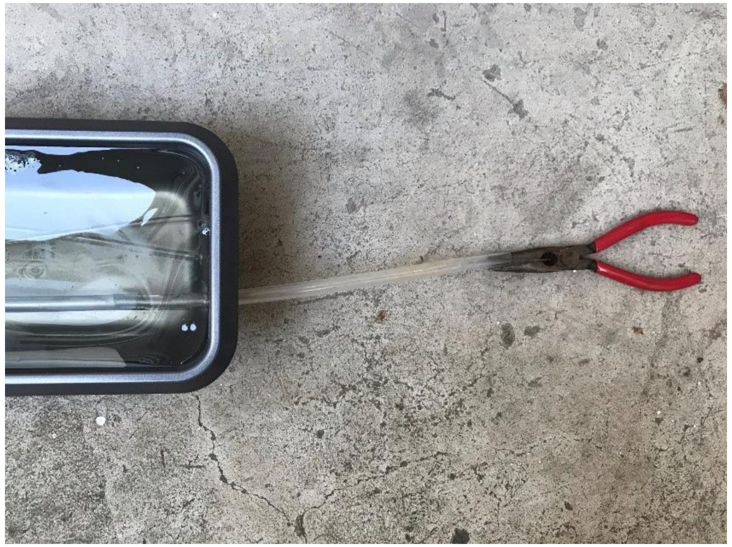

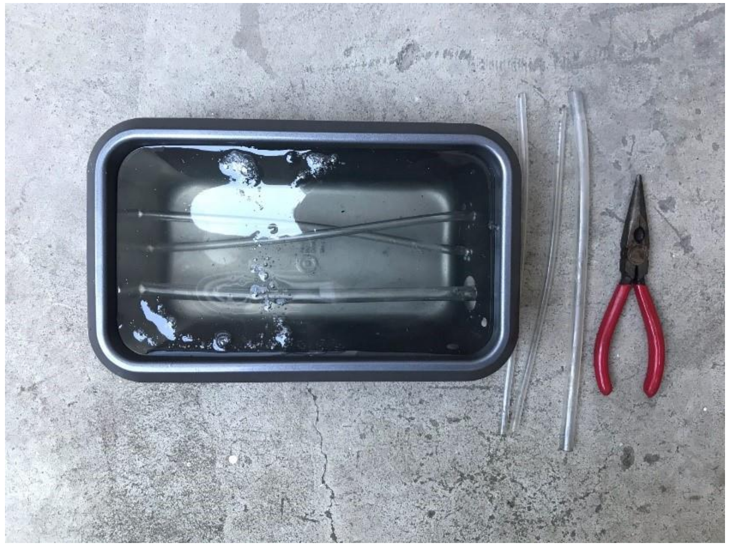

9. Remove tubing with slow steady traction and slight rotation

Author-Owned Images

10. Lift sides of mold from edges and flip pan over. If gel remains stuck, gently lift out from sides. It is still unlikely to tear.

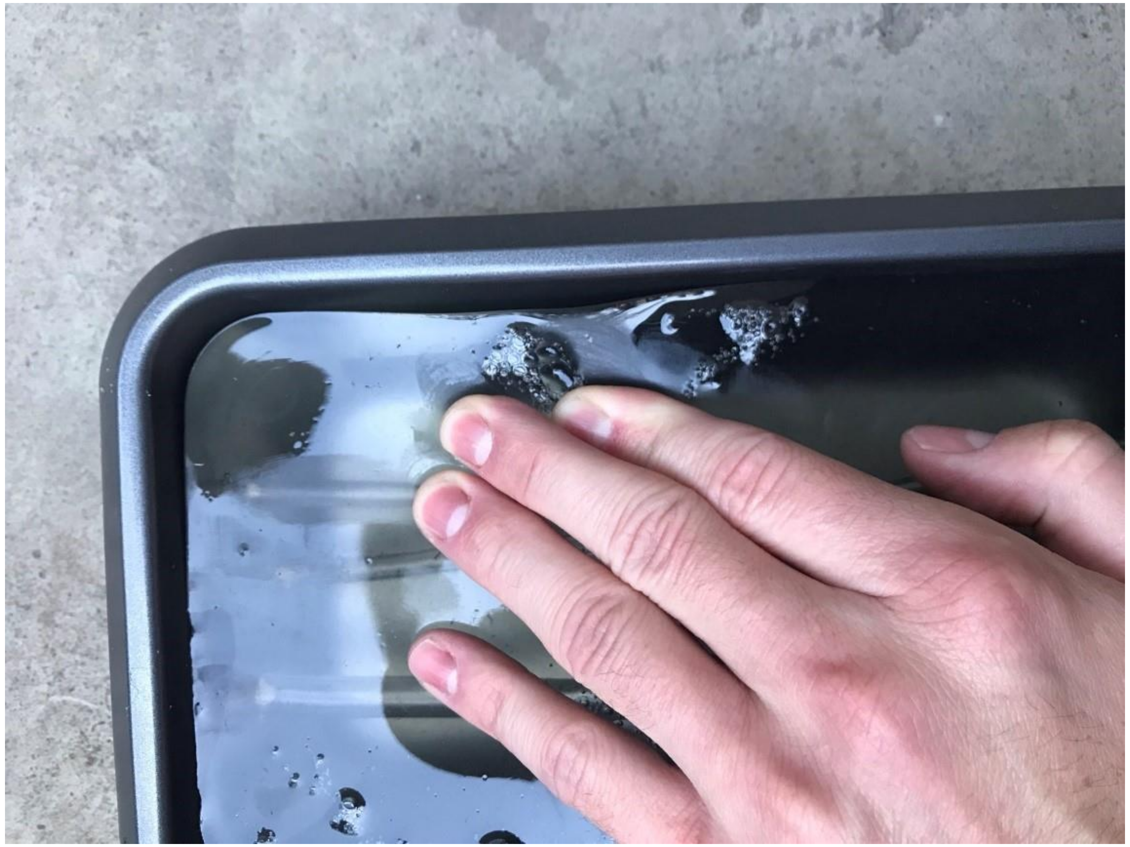

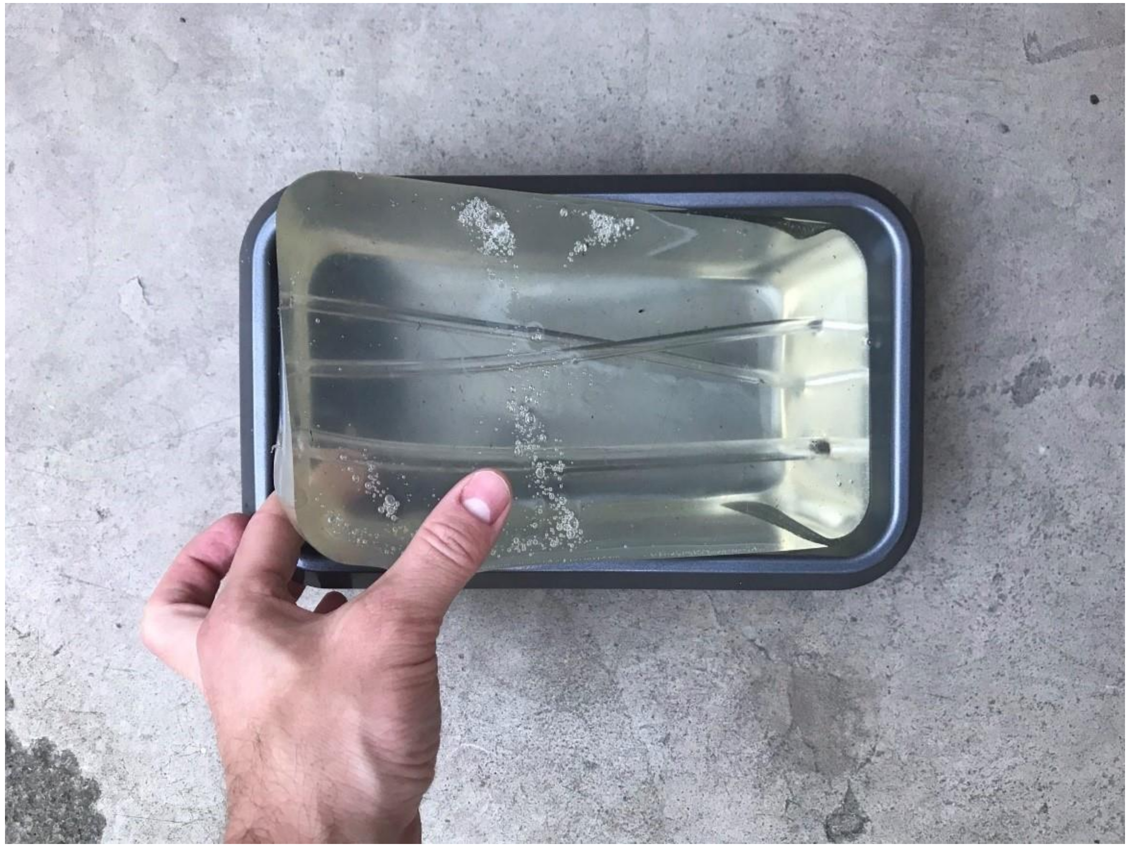

Author-Owned Images

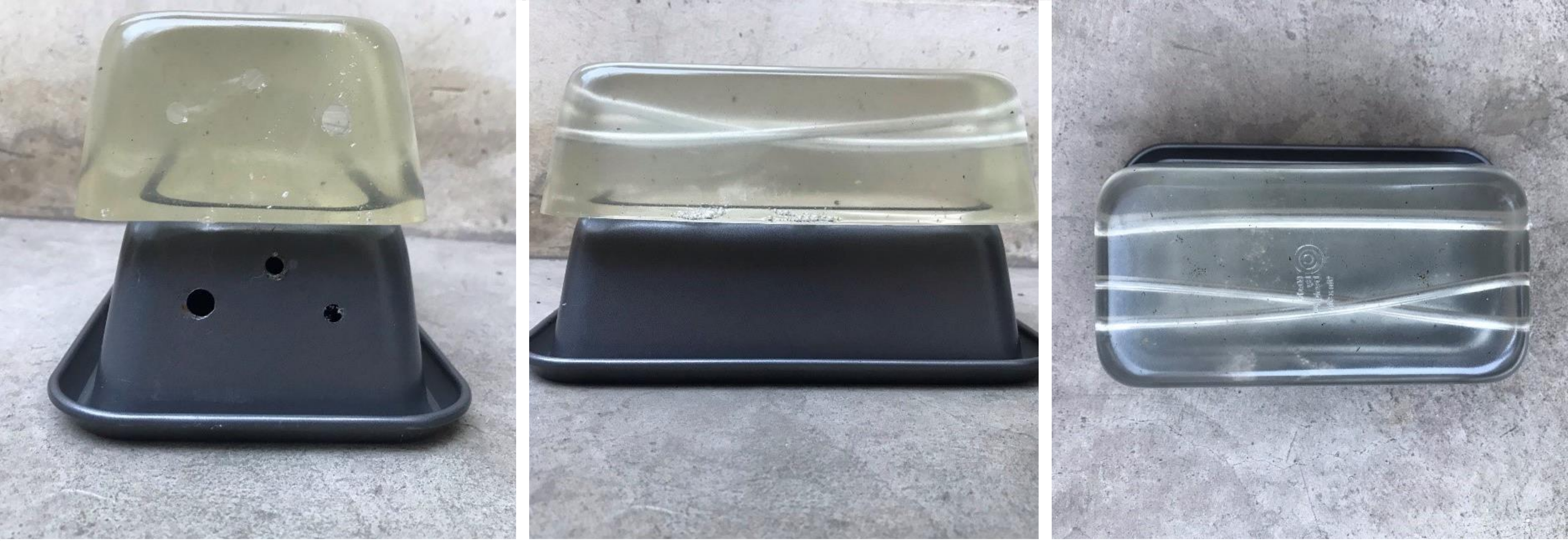

11. Inspect completed gel

Author-Owned Images

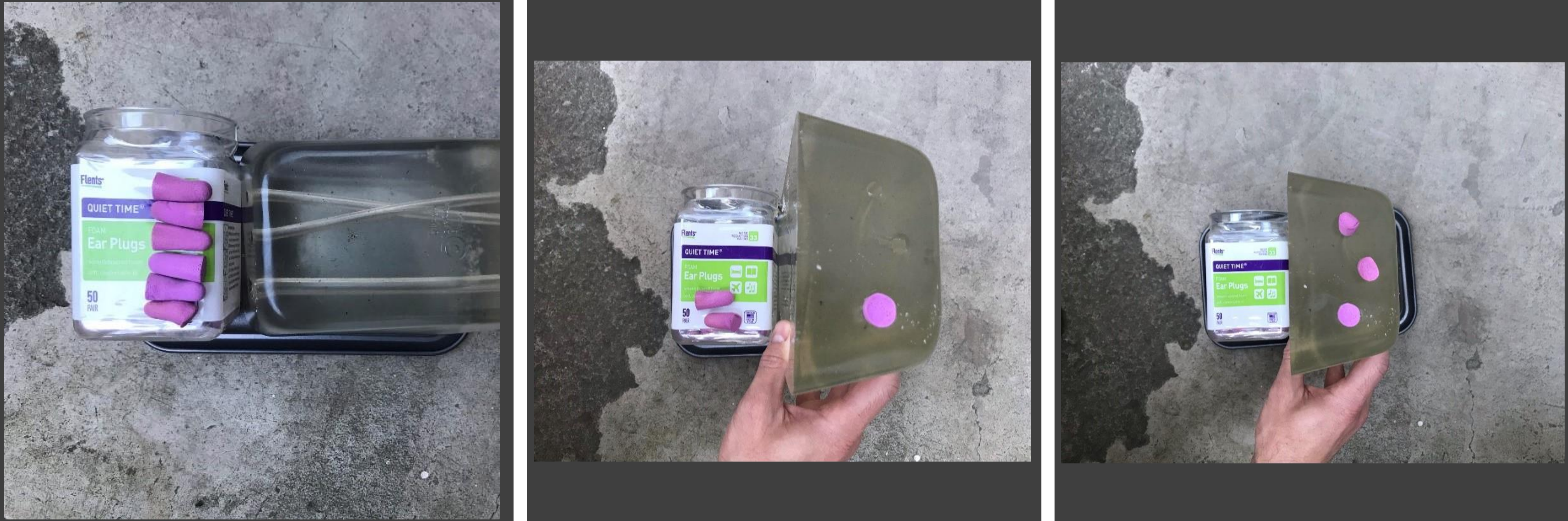

12. Place earplugs into one side of empty “veins”.

Author-Owned Images

13. Fill “veins” with water. Place second set of earplugs on the other side of the “veins” to seal. Gel model is now complete!

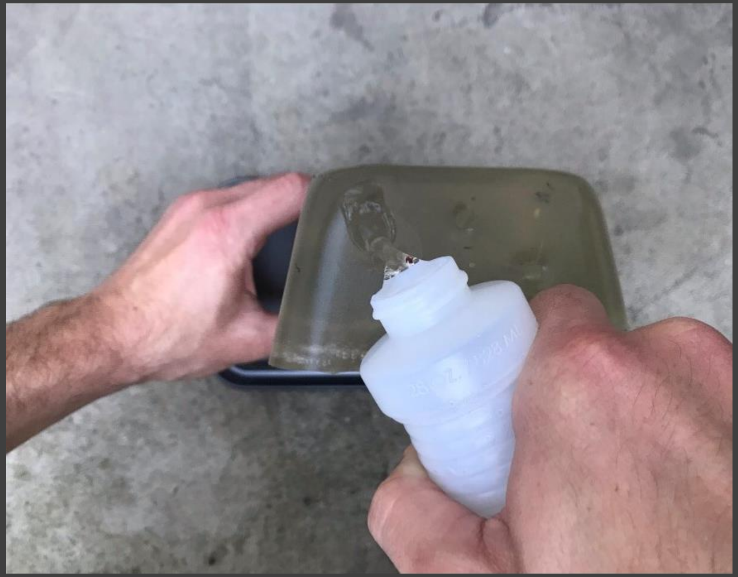

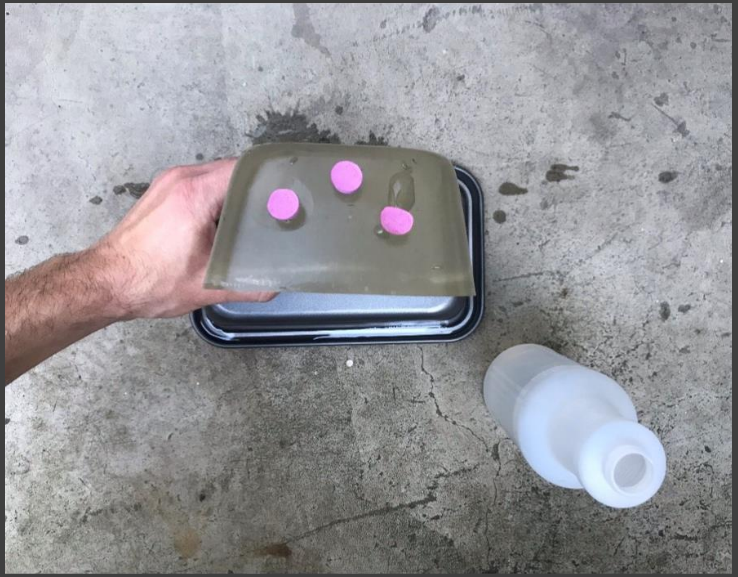

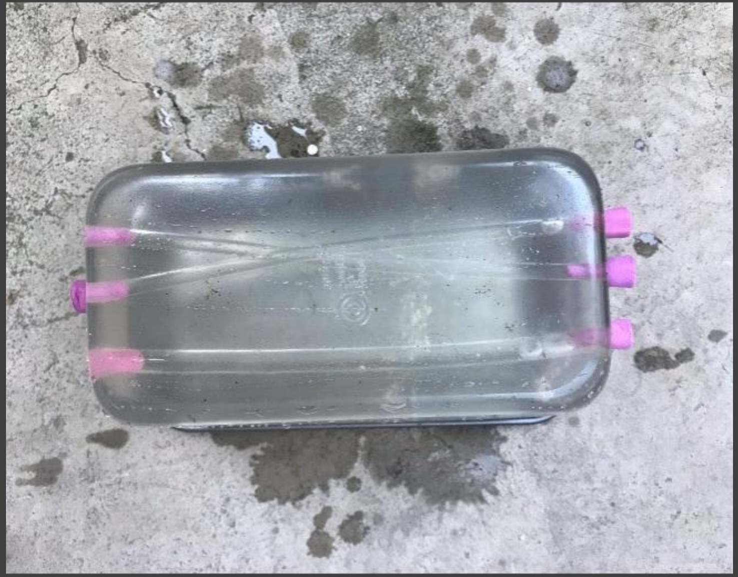

Author-Owned Images
