# Supplementary material for: Teaching Module on Ultrasound-Guided Venous Access Using a Homemade Gel Model for Fourth-Year Medical Students
Source: MedEdPORTAL. 2022 Feb 2;18:11222. doi: 10.15766/mep_2374-8265.11222 (PMC8807663; doi:10.15766/mep_2374-8265.11222)
Supplement: Supplementary file 1 — Ultrasound-Guided Peripheral Venous Access.mp4Practical Session Room Setup.pdfSmall-Room Setup.docxPhoto Deck Directions.pdfItemized Materials for Creating Gel Models.docxFacilitator Guide.docxSchedule.docxPremodule Survey.docxPostmodule Survey.docxDirectly Observed Procedural Skills Evaluation.docx [file mep_2374-8265.11222-s001.zip › E. Itemized Materials for Creating Gel Models.docx]

**Appendix E: Itemized Materials for Creating Gel Models**

Specific brands are listed; other brands could be substituted without disadvantage.

1. Ballistic Gel

Clear Ballistics | $42.80 for 20% Gelatin Block 18x4x4 in

2. Slow Cooker

Crock-Pot | $40.50

3. Griddle

Presto | $34.83

4. Bread Pan

Wilton | $8.39

5. Scissors

Westcott | $5.74

6. Needle-Nose Pliers

Sea Striker | $8.00

7. Tubing Small (1/4 in outer diameter) Vinyl

Everbilt | $0.92/ft

8. Tubing Large (3/8 In Outer Diameter) Vinyl

Everbilt | $0.33/ft

9. Drill

Ryobi | $49.00

10. Drill Bit Set

Ryobi | $7.97

11. Foam Earplugs

Flents | $13.99

12. Heat-Resistant Gloves/Mitts

Kitchen Perfection | $26.49

13. Eye Protection

Super More | $12.99
