# Supplementary material for: Teaching Module on Ultrasound-Guided Venous Access Using a Homemade Gel Model for Fourth-Year Medical Students
Source: MedEdPORTAL. 2022 Feb 2;18:11222. doi: 10.15766/mep_2374-8265.11222 (PMC8807663; doi:10.15766/mep_2374-8265.11222)
Supplement: Supplementary file 1 — Ultrasound-Guided Peripheral Venous Access.mp4Practical Session Room Setup.pdfSmall-Room Setup.docxPhoto Deck Directions.pdfItemized Materials for Creating Gel Models.docxFacilitator Guide.docxSchedule.docxPremodule Survey.docxPostmodule Survey.docxDirectly Observed Procedural Skills Evaluation.docx [file mep_2374-8265.11222-s001.zip › G. Schedule.docx]

**Appendix G: Schedule**

Session Schedule

- A: 8:00-8:30 am  (25 students)
- B: 8:40-9:10 am  (25 students)
- C: 9:20-9:50 am  (25 students)
- D: 10:00-10:30 am  (25 students)
- E: 10:40-11:10 am  (25 students)
- F: 11:20-11:50 am  (25 students)

Station Assignments for Each of the Six Sessions (A-F) – Clinical Skills Center

- Room 5 (5 students) Resident
- Room 6 (5 students) Resident
- Room 7 (5 students) Resident
- Room 8 (5 students) Resident
- Room 9 (5 students) Resident

Alternates/Rotators:

--Attending (pit-boss)

--Attending (pit-boss)
