# Supplementary material for: Teaching Module on Ultrasound-Guided Venous Access Using a Homemade Gel Model for Fourth-Year Medical Students
Source: MedEdPORTAL. 2022 Feb 2;18:11222. doi: 10.15766/mep_2374-8265.11222 (PMC8807663; doi:10.15766/mep_2374-8265.11222)
Supplement: Supplementary file 1 — Ultrasound-Guided Peripheral Venous Access.mp4Practical Session Room Setup.pdfSmall-Room Setup.docxPhoto Deck Directions.pdfItemized Materials for Creating Gel Models.docxFacilitator Guide.docxSchedule.docxPremodule Survey.docxPostmodule Survey.docxDirectly Observed Procedural Skills Evaluation.docx [file mep_2374-8265.11222-s001.zip › F. Facilitator Guide.docx]

**Appendix F: Facilitator’s Guide for Hands-on Instruction**

**Summary of Tips**

Teaching

- Your role is to guide the student to independently achieve intravenous access with “blood return.” Each student will have allotted a maximum of 5 minutes.
- The students will be asked to watch a 5-minute instructional video beforehand. As a refresher, you should first show each new student group how to hold the transducer and needle at the beginning of each session.
- Prompt students to complete the checklist items in the Direct Observation of Procedural Skills Evaluation (DOPSE).
- You should hold the iPad in the correct line of sight for the student. Change the depth and gain as needed.
- See additional general instructions, goals and objectives, resources, and teaching guidance below

Gel Model Usage

- The gel is sturdy, but a needle attempt will leave a small residual track visualized on the US screen. Thus, the attempts need to be spaced out on the gel surface so that each student gets to attempt the procedure on an untouched region of gel.
- Each gel model has 3 “vessels” of varying depths. The gel is long enough such that, for each vessel, one student can attempt the procedure on each half of the gel. That means that 6 students can perform the procedure on each side of the gel (ie 3 students on the left, 3 students on the right).
- The gel can then be flipped upside down, and 6 more students can perform the procedure as above.
- After each student aspirates “blood” from the “vessel”, make sure he/she re-injects the “blood” slowly so that there will be enough for subsequent students to attempt this aspiration. If needed, there will be saline flushes (or tap water) available to replenish the “blood.”

Assessment and Data Recording

- Complete each student’s DOPSE form assessment (Appendix J) immediately after his/her venous access attempts (approximately within 5 minutes). Leave these in a pile by your station. They will be collected at the end of each 30-minute session.
- The US Director will dispense and collect both the Pre- and Post-Module Surveys.
- The students will not be recorded as present at this mandatory session unless these forms are submitted.

**Additional Faculty Development/Facilitator Guidance:**

General instructions:

- Please note that the Direct Observation of Procedural Skills session for ultrasound guided IV placement includes multiple steps. The following information will assist you in preparing for a successful session.
- Review the didactics and DOPSE forms prior to arrival (insert links on how to access here). Please note that you may have learned or perfected several techniques to assist you but for this session we will follow a standardized uniform format as discussed in student didactic. This will ensure comparability and standardization of assessment across groups.
- Arrive at least 20 minutes before your listed start of session. This will enable us to hold an on-site orientation and run through of the day and format and will allow for answering of any clarifying questions you may have.
- All facilitators for the day will have a 5 minute group practice session for training on student rating. We will role play a student performing the procedure on the model and each facilitator will also complete checklist. We will compare the ratings and debrief in order to standardize form completion.
- We recommend that each facilitator tests the individual room equipment and performs the procedure on task trainer once, prior to session with students, in order to troubleshoot for equipment or gel/checklist issues.

Overall curriculum details:

We will be using the Kolb’s experiential cycle of watching, thinking, doing, and reflecting to facilitate this session.
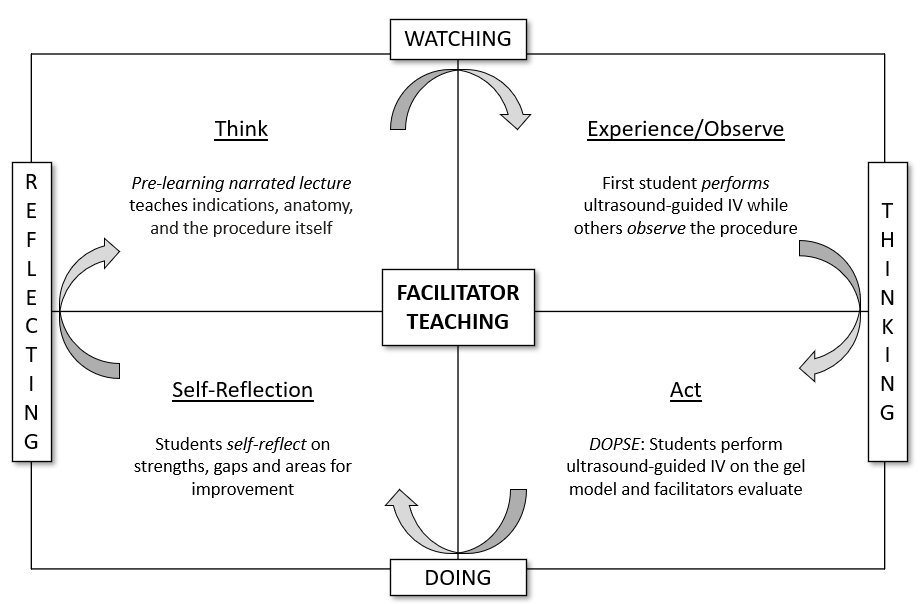


Author-Owned Figure

**Steps to the Curriculum:**

1. Didactics: Pre-learning narrated lecture

Students will receive didactic information (lecture, video, anatomy, indications, and procedure steps) prior to coming to your assigned room. Facilitators should preview this lecture (provide instructions for access) to familiarize themselves of content. Please note we require each facilitator to review this content regardless of your own skill proficiency in ultrasound guided IV access skills. The didactic will set the standards for what is expected of students for this skill assessment and to ensure comparability of teaching and assessment across groups.

2. In session

Please spend a few minutes reinforcing concepts such as how to hold transducer and questions from students.

Encourage peer queries and allow for students to practice, time allowing. Students should be observing while they are not performing task themselves. (You may want to ask for peer feedback at end of session).

3. DOPSE: Complete checklist while student performs procedure. Please familiarize yourself with the DOPSE form and checklist elements prior to session.

4. Debrief with students, what went well, what would they do differently next time and allow for questions and/or peer feedback.

**Additional Resources**

1. Walker M, Peyton JWR. Teaching in the theatre. In: Peyton JWR, editor. *Teaching and Learning in Medical Practice*. Rickmansworth: Manticore Publishers Europe Ltd; 1998:171–180.
2. Nikendei C, Huber J, Stiepak J, et al. Modification of Peyton's four-step approach for small group teaching - a descriptive study. *BMC Med Educ*. 2014;14:68. Published 2014 Apr 2. doi:10.1186/1472-6920-14-68.
3. Wilkinson JR, Crossley JG, Wragg A, Mills P, Cowan G, Wade W. Implementing workplace-based assessment across the medical specialties in the United Kingdom. *Med Educ*. 2008;42(4):364-373. doi:10.1111/j.1365-2923.2008.03010.x.
4. Osborn SR, Borhart J, Antonis MS. Medical students benefit from the use of ultrasound when learning peripheral IV techniques. *Crit Ultrasound J*. 2012;4(1):2. Published 2012 Mar 6. doi:10.1186/2036-7902-4-2.
5. Vusse LV, Shepard A, Bergam B, Andros J, Morris A. Procedure training workshop for internal medicine residents that emphasizes procedural ultrasound: logistics and teaching materials. *MedEDPORTAL*. 2020. doi.org/10.15766/mep_2374-8265.10897.
6. Vitto MJ, Myers M, Vitto CM, Evans DP. Perceived difficulty and success rate of standard versus ultrasound-guided peripheral intravenous cannulation in a novice study group. *J Ultrasound Med*. 2016;35:895-898. doi:10.7863/ultra.15.06057.
