# Supplementary material for: Teaching Module on Ultrasound-Guided Venous Access Using a Homemade Gel Model for Fourth-Year Medical Students
Source: MedEdPORTAL. 2022 Feb 2;18:11222. doi: 10.15766/mep_2374-8265.11222 (PMC8807663; doi:10.15766/mep_2374-8265.11222)
Supplement: Supplementary file 1 — Ultrasound-Guided Peripheral Venous Access.mp4Practical Session Room Setup.pdfSmall-Room Setup.docxPhoto Deck Directions.pdfItemized Materials for Creating Gel Models.docxFacilitator Guide.docxSchedule.docxPremodule Survey.docxPostmodule Survey.docxDirectly Observed Procedural Skills Evaluation.docx [file mep_2374-8265.11222-s001.zip › J. Directly Observed Procedural Skills Evaluation.docx]

**Appendix J: Directly Observed of Procedural Skills Evaluation (DOPSE)**

Student:  ____________________

Faculty Observing:  ____________________

Date:  ____________________

| **Skill** | **Requires verbal prompting with performance errors** | **Works Independently with minor errors** | **Works independently without errors** |
| --- | --- | --- | --- |
| Orients the transducer marker properly (left side of screen corresponds to left side of gel model). |  |  |  |
| Places the ultrasound on the gel model in transverse plane such that the vein is located in the center of the screen. |  |  |  |
| Uses the proper angle of needle insertion in relation to vessel depth (30-45 degrees). |  |  |  |
| Maintains visualization of the needle tip as it advances towards the vessel. |  |  |  |
| Visualizes the needle within the vessel in the longitudinal plane. |  |  |  |
| Aspirates “blood” return from the vessel and re-inserts it. |  |  |  |
| Number of separate needle puncture attempts to achieve success:  ð 1                               ð 2                              ð 3                               ð >3 | | | |
| In his/her group of 5-6 students, this student attempted the procedure:  ð First                   ð Second                  ð Third                   ð Fourth                  ð Fifth                   ð Sixth | | | |

| **Global Rating (Please check one):** | |
| --- | --- |
|  | *Trusted to observe only |
|  | *Trusted to perform with direct supervision and coaching |
|  | *Trusted to perform with indirect supervision |
|  | *Trusted to perform without supervision |

**Since entrustment includes deliberate practice and often multiple assessments over time, the Global Rating scale should be modified as follows: At the end of this session the learner is able to: 1) Observe only, 2) Perform this skill with direct observation and coaching, 3) Perform this skill with indirect supervision, and 4) Perform this skill without supervision*
